# Concerns about the paper "A descriptive study of culture media in Brazilian assisted reproduction clinics"

**DOI:** 10.5935/1518-0557.20170014

**Published:** 2017

**Authors:** Hubert Joris, Christer Silversand

**Affiliations:** 1 Vitrolife Sweden - Göteborg, Sweden

Dear Editor,

With great interest we read the article in the recent issue of JBRA by Bartmann *et al.* (2016) on the use of culture media by IVF clinics in Brazil.

Besides the information on current media usage by IVF laboratories, Bartmann *et al.* (2016) also list the components from the media currently in use. In the media profiles section, the authors provide information about the components in the different media used in IVF laboratories in Brazil. It appears to us that this section has limited information, justifying a rectification to avoid confusion for the readers.

There is a lot of information and discussions available on the development of modern culture media and its components. Specifically for the G-series media, the full composition of an earlier version of the media has been published (Lane *et al.*, 2003). More recently, Morbeck *et al.* (2014a) published an analysis on the composition of commercial culture media, including various of the media discussed in the paper by Bartmann *et al.* (2016). Additionally, specifically for Vitrolife media, the medium components used are available for customers on the lot-specific certificate of analysis included in every media shipment.

In general, the authors state that information is missing for certain products or is unclear about the presence of components and cannot, therefore, be listed. It is unknown to us if the authors contacted medium producers to obtain more detailed information. Considering the availability of all this information, we were very surprised about the missing information on Vitrolife and other manufacturters' culture media composition. For relevant papers such as the study by Bartmann *et al.* (2016) Vitrolife is willing to provide useful information.

Culture medium is a crucial item in an IVF treatment. We feel that it is important to provide correct information and hereby would like to add important information not provided by Bartmann *et al*. (2016).

The authors state: "only 3 media have dipeptides in their composition ...". Vitrolife introduced a more stable form of glutamine in culture media very early on (Lane *et al.* 2003). Since then, the use of a more stable form of glutamine has gradually been introduced by most medium manufacturers. It has been shown in a number of studies that ammonium can affect embryo development; and very recently it was shown that, compared to a more stable dipeptide, the presence of glutamine results in very high ammonium levels during incubation as well as during storage (Kleijkers *et al.* 2016). The effect of ammonium levels on embryo development has also been demonstrated in humans (Virant-Klun *et al*., 2006).

Another statement in this section is: "Antioxidants are present just in the cleavage medium..." Several components in the culture media play the role of antioxidant. The role of EDTA in culture media to overcome 2-cell block was described many years ago (Abramczuk *et al*., 1977) and today it is present in most modern culture media. The embryo culture medium from Vitrolife also contains sodium citrate besides EDTA, the medium for culture of cleavage-stage embryos contains lipoic acid.

The authors also state: "As far as energy substrates are concerned, only Vitrolife had just one type (hyaluronan);...". All media for culture of embryos contain energy sources such as pyruvate and lactate. These are fundamental components in media for embryo culture. There has been discussions on the requirements for glucose, but today there is consensus that glucose is required and it is present in modern culture media. More specifically for the G-series media, the levels of energy sources required for embryo culture at the cleavage stage and blastocyst stage were determined based on measurements in human oviducts, and uterine fluids collected at relevant time points of the menstrual cycle (Gardner *et al*., 1996). Additionally, hyaluronan is a macromolecule with different functions but its primary role is not to act as an energy source.

Regarding albumin, Bartmann *et al*. (2016) stated the following: "...product containing human originated protein in their composition have the potential presence of contaminants sourced from such obscure nature protein." This statement may be interpreted that there is a difference in quality when media are not yet supplemented with protein. We agree with Bartmann *et al.* (2016) that human albumin contains a number of undefined components (Dyrlund *et al*. 2014). However, albumin used in culture media for IVF undergoes very strict testing, maximizing safety and it is approved for use in human IVF by regulatory bodies. Additionally, albumin used for supplementation provided by the same manufacturer is from the same source, and undergoes the same extensive testing and regulatory approval. It has also been shown that albumin may have an effect on mouse embryo development (Morbeck *et al*., 2014b). It is the manufacturers' responsibility to test raw materials sufficiently so that any potential negative effect on human embryo development is ruled out. However, when clinics are using other sources of albumin than those regulatory approved for IVF, performance and safety of the product cannot be guaranteed and it becomes the users' responsibility.

A final statement we would like to comment on is: "Salt and ions are present in most of them, but in case of Vitrolife...". Ions are very important in any culture media. They have important roles in different processes but are also the major contributor for obtaining the correct osmotic pressure that is crucial for proper development. As stated earlier, information about all components and thus also ions used in media from Vitrolife is available through different sources.

For the sake of completeness, we hereby list in alphabetical order the components of Vitrolife media referred to by Bartmann *et al.* (2016):

G-1: alanine, alanyl-glutamine, asparagine, aspartate, calcium chloride, EDTA, gentamicin, glucose, glutamate, glycine, hyaluronan, lipoic acid, magnesium sulphate, methionine, potassium chloride, proline, serine, sodium bicarbonate, sodium chloride, sodium citrate, sodium dihydrogen phosphate, sodium lactate, sodium pyruvate, taurine and water

G-2: alanine, alanyl-glutamine, arginine, asparagine, aspartate, calcium chloride, calcium pantothenate, cystine, gentamicin, glucose, glutamate, glycine, histidine, hyaluronan, isoleucine, leucine, lysine, magnesium sulphate, methionine, phenylalanine, potassium chloride, proline, pyridoxine, riboflavin, serine, sodium bicarbonate, sodium chloride, sodium citrate, sodium dihydrogen phosphate, sodium lactate, sodium pyruvate, thiamine, threonine, tryptophan, tyrosine, valine and water G-1 PLUS and G-2 PLUS also contain human serum albumin.

Studies such as the paper by Bartmann *et al*. (2016) are important to provide users with valuable information about products used in their daily practice. However, we felt important information was missing and hoped to add to the readers' knowledge about the composition of culture media.
